# Strategies of exploitation of mammalian reservoirs by *Bartonella* species

**DOI:** 10.1186/1297-9716-43-15

**Published:** 2012-02-27

**Authors:** Hongkuan Deng, Danielle Le Rhun, Jean-Philippe R Buffet, Violaine Cotté, Richard J Birtles, Muriel Vayssier-Taussat

**Affiliations:** 1 USC INRA Bartonella et Tiques, ANSES, 23 Avenue du Général de Gaulle, 94700, Maisons-Alfort, France; 2 School of Environment and Biological Sciences, University of Salford, Salford, Greater Manchester, M5 4WT, United Kingdom; 3 University of Texas San Antonio, One UTSA Circle, San Antonio, TX 78249, USA

## Abstract

Numerous mammal species, including domestic and wild animals such as ruminants, dogs, cats and rodents, as well as humans, serve as reservoir hosts for various *Bartonella* species. Some of those species that exploit non-human mammals as reservoir hosts have zoonotic potential. Our understanding of interactions between bartonellae and reservoir hosts has been greatly improved by the development of animal models for infection and the use of molecular tools allowing large scale mutagenesis of *Bartonella* species. By reviewing and combining the results of these and other approaches we can obtain a comprehensive insight into the molecular interactions that underlie the exploitation of reservoir hosts by *Bartonella* species, particularly the well-studied interactions with vascular endothelial cells and erythrocytes.

## 

Table of contents

1 Introduction

2 Dynamics of infection in mammalian reservoir hosts

3 Step 1: Infection prior to bacteraemia

3.1 The BadA/Vomp/Brp adhesions

3.2 The VirB/D4 type IV secretion system and its effector proteins

4 Step 2: Seeding of blood and extra cellular survival

5 Step 3: Erythrocyte encounter and adhesion

6 Step 4: Invasion of and persistence within erythrocytes

7 Conclusions

8 Competing interests

9 Author’s contributions

10 Acknowledgments

11 References

## Introduction

*Bartonella* species are small, curved, pleomorphic, fastidious, haemotropic Gram-negative bacteria that have specifically adapted to infect mammals. Although 26 *Bartonella* species or sub-species have been formally validated to date, many more, as yet partially characterized, await formal proposal. Bartonellae have been encountered in mammals all over the world, however the prevalence of infections and their public health and veterinary importance vary according to species and geographical region.

*Bartonella* species are transmitted between mammalian hosts by blood-feeding arthropods. Numerous, diverse arthropods have been implicated in the transmission of bartonellae, but each *Bartonella* species appears to exploit only one, or few, arthropod species. The role of arthropods in the natural cycle of bartonellae may extend beyond mere vectors; there is some evidence to support their role as additional reservoirs for the bacteria. Each *Bartonella* species also appears to be highly adapted to one or few mammalian reservoir hosts [1,2] in which they establish a long-lasting intra-erythrocytic bacteraemia as the hallmark of infection [3]. This bacteraemia does not appear to cause immediate detriment to the host. In general, bartonellae provoke acute clinical manifestations only when accidentally introduced into the wrong host or when encountering immunocompromised individuals among reservoir populations.

**Figure 1:**
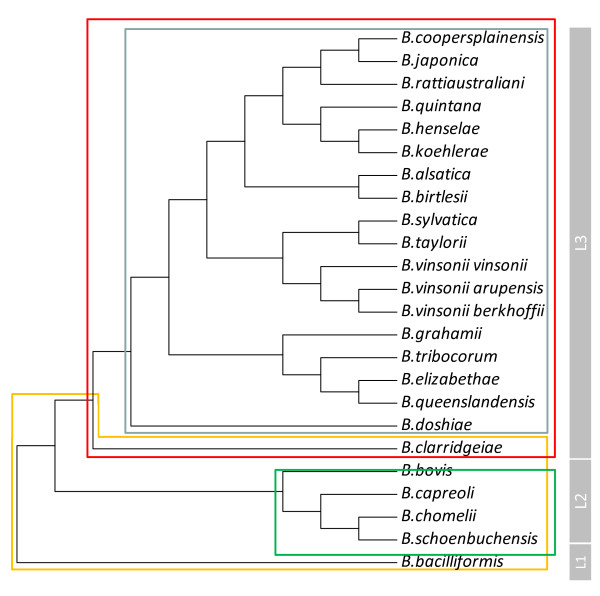
**Molecular phylogenetic analysis of the 24 extant, validated* Bartonella *taxa inferred from alignment of partial (326 bp)* gltA *sequences.** Evolutionary history was inferred by using the Maximum Likelihood method based on the Tamura-Nei model. The tress with the highest log likelihood is shown. Evolutionary analyses were conducted in MEGA5. Imposed on the tree are the distributions of the three T4SSs (red box = VirB/D4, blue box = Trw, green box: Vbh) and flagella (orange box) amongst the taxa. To the right of the tree is an indication of the three evolutionary lineages defined within the genus.

The *Bartonella* genus lies among the alpha proteobacteria in proximity to the genus *Brucella*. Both genera are classified in the family *Rhizobiales* that also embraces a large number of taxa of plant-associated and environmental bacteria. Twenty-four *Bartonella* species have been validly described to date, with one species, *Bartonella vinsonii*, subdivided into three subspecies (Table 1). Inference of the phylogenetic relationships within the genus reveals the profound divergence of *Bartonella bacilliformis*, which lies alone on an “ancient” ancestral lineage (lineage 1), apart from the other *Bartonella* species. The remaining “modern” species form two further lineages, with lineage 2 containing the four ruminant-associated species (*Bartonella bovis, Bartonella capreoli, Bartonella chomelii and Bartonella schoenbuchensis*), and lineage 3 containing the remaining 19 species (Figure 1). Within lineage 3, *Bartonella clarridgeiae* is the outlier, and phylogenetic studies that have also included as yet only partially characterised bartonellae have suggested that this species is a representative of a fourth lineage within the genus [4,5]. The complete genome sequences of six *Bartonella* species (*B. bacilliformis, B.clarridgeiae, Bartonella grahamii, Bartonella henselae, Bartonella quintana* and *Bartonella tribocorum*) have been published to date, although efforts to sequence the genomes of all remaining species and numerous partially-characterised bartonellae are also underway. The sizes of the published genomes range from 1.45 Mb to 2.62 Mb [4,6].

**Table 1:** Validated *Bartonella* species, their reservoir hosts, and their currently perceived medical relevance

*** Bartonella *species**	**Proven/suspected reservoir host**	**Evidence of human infections?**
*B.alsatica*	rabbits	yes
*B. bacilliformis*	humans	yes
*B. birtlesii*	small rodents	
*B.bovis*	ruminants	
*B. capreoli*	ruminants	
*B. chomelii*	ruminants	
*B. clarridgeiae*	felids	yes
*B. coopersplainensis*	rats	
*B. doshiae*	small rodents	
*B. elizabethae*	rats	yes
*B. grahamii*	small rodents	yes
*B. henselae*	felids	yes
*B. japonica*	small rodents	
*B. koehlerae*	felids	yes
*B.peromysci*	small rodents	
*B. queenslandensis*	rats	
*B. quintana*	humans	yes
*B. rattaustraliani*	rats	
*B. schoenbuchensis*	ruminants	
*B. silvatica*	small rodents	
*B. talpae*	moles	
*B. taylorii*	small rodents	
*B. tribocorum*	rats	yes
*B. vinsonii subsp. arupensis*	small rodents	yes
*B. vinsonii subsp. berkhoffii*	canids	yes
*B. vinsonii subsp. vinsonii*	small rodents	yes

*B. bacilliformis* and *B. quintana* are the two *Bartonella* species that appear to exploit humans as reservoir hosts (Table 1). Infections with *B. bacilliformis* only occur in the Andean region of South America [7,8], and this geographical distribution correlates with the range of the *Lutzomyia* species that are thought to transmit infections [8]. *B. bacilliformis* appears to be potentially the most pathogenic *Bartonella* species, provoking a remarkably bi-phasic disease referred to as bartonellosis. Acute bartonellosis manifests as Oroya fever, a disease which is characterized by a severe haemolytic anaemia effecting, in some cases, nearly 100% of erythrocytes. Fatality rates of up to 80% have been described in patients not receiving antibiotic treatment [9]. *B. bacilliformis* infections can also manifest as verruga peruana, characterized by vascular tumours that result from the massive proliferation of endothelial cells and which can persist for more than one year [7,8]. Verruga peruana was probably recognized during pre-Columbian times, but its aetiology was not resolved until 1905, when *B. bacilliformis* was first isolated. *B. quintana* infections were first recognized during World War I in the form of trench fever (or five-day fever), but the bacterium was not isolated until 1961. Medical interest in *B. quintana* waned after World War II as infections became rare, but, at the end of the twentieth century, *B. quintana* re-emerged as a bacterium of public health importance with recognition of its role in persistent bacteraemia in the homeless, drug and alcohol addicts (“urban” trench fever) and refugees. Infection is usually characterized by a chronic intra-erythrocytic bacteraemia with few, mild, self-limiting symptoms [10], although more severe manifestations such as endocarditis and bacillary angiomatosis have also been reported [10]. *B. quintana* is transmitted by the human body louse (*Pediculus humanus humanus*), an ectoparasite that is specific to humans but which only emerges when clothes remain unchanged and unwashed [11]. Thus, as with *B. bacilliformis*, vector behaviour is a key determinant in the epidemiology of *B. quintana* infections.

Numerous domestic and wild animals, including ruminants, felids, canids and rodents serve as reservoir hosts for various *Bartonella* species (Table 1) [2]. To date, evidence of zoonotic potential has been reported for 10 of these species (Table 1). In these reports, no evidence of an intra-erythrocytic presence of zoonotic bartonellae in infected humans has been reported [12]. Of the zoonosis-associated *Bartonella* species, *B. henselae* is the most frequently associated with human disease. *B. henselae* exploits felids, including domestic cats as reservoir hosts, between which infection is transmitted by cat fleas (*Ctenocephalides felis*) [13]. As is typical for reservoir hosts, cats usually do not develop any apparent symptoms of infection, which can persist for months or years [14]. *B. henselae* transmission from cats to humans is thought to occur primarily by the inoculation of infected flea faeces via cat scratches or bites [13], although there is also some evidence that infection can be acquired from ticks [15,16]. *B. henselae* can provoke a wide variety of clinical manifestations in humans, including, most commonly, lymphadenopathy (cat scratch disease), malaise, fever and splenomegaly that can persist for several months. Less frequently (< 25% of cases) [17], more serious manifestations can occur, including angiomatosis (in immunocompromised patients), ocular disorder, encephalitis, meningitis, glomerulonephritis and endocarditis [12].

The other nine zoonosis-associated *Bartonella* species exploit a range of reservoir hosts; for example, *B. vinsonii* subspecies *berkhoffii* parasitizes canids (domestic dogs, coyotes and gray foxes) and *Bartonella alsatica* parasitizes rabbits. Others, such as *B. grahamii* are associated with woodland rodents (Table 1). In addition to their potential as human pathogens, numerous *Bartonella* species have been implicated in veterinary infectious diseases, including, most frequently, *B. henselae* and *B. vinsonii* subspecies *berkhoffii*. Many of the manifestations observed in cats and dogs are akin to those observed in humans.

## Dynamics of infection in mammalian reservoir hosts

The life cycle of bartonellae in their reservoir hosts has been deduced through observations of naturally infected mammals and through experimental infections. In a seminal study [3], the evolution of infection of *B.tribocorum* in laboratory rats was monitored using a green fluorescent protein (GFP)-tagged bacterial strain in conjunction with microscopy and flow cytometry. This study found that, following intravenous injection (models incorporating a natural route of infection still remain generally elusive), bacteria could be initially detected in the blood but were soon cleared, only to reappear again between two and five days later. Only on reappearance were bartonellae observed inside erythrocytes, although only a small proportion of erythrocytes were infected. Erythrocytes were usually invaded by a single bacterium, which, once inside, replicated in a membrane-bound compartment over a period of several days until, on average, eight daughter cells were created. Replication then ceased and infected erythrocytes were shown to persist in circulation for several weeks. This process did not provoke symptoms in the infected rat [3]. Experiments using other animal models, such as a *B. birtlesii*-mouse model [18-20], and a *B. henselae*-cat model [21] have yielded results that are akin to those observed in the *B. tribocorum*-rat model, suggesting a common infection mode for all *Bartonella* species in their respective animal reservoirs [22]. Furthermore, the kinetics of bacteraemia observed in these models were similar to those observed in captured naturally-infected animals (unpublished observations), which supports the reliability of results obtained from artificial routes of inoculation.

Our understanding of bartonellae/host interactions has been greatly improved by the development of molecular tools allowing large scale mutagenesis of *Bartonella* species such as *B. tribocorum* and *B. birtlesii*. The use of these tools in conjunction with judicious screening in relevant animal models (rats and mice respectively) has resulted in the identification of numerous genetic sequences, the integrity of which are necessary for the establishment of bacteraemia by bartonellae [4,19]. Those genetic sequences can be classified into six groups: (1) genes previously implicated in *Bartonella* infection of its mammalian hosts, (2) genes encoding cell envelope components, (3) genes encoding proteins involved in metabolism, (4) phage-related genes, (5) genes encoding proteins of unknown function and (6) intergenic regions. These data, when combined with the results of in vitro studies, allow us to piece together an overview of the molecular basis of reservoir host exploitation by *Bartonella* species.

## Step 1: Infection prior to bacteraemia

As mentioned above, inoculation of a susceptible host appears to be primarily mediated by the introduction of infected vector faeces into cuts or scratches on the skin. However, the fate of infecting bacteria immediately following inoculation, prior to their appearance in the bloodstream, remains uncertain. Nonetheless, it is clear that bartonellae can colonise highly vascularised tissues like the liver and spleen during the first days of infection, as well as the vascular bed of the skin (for *B. bacilliformis*) [23]. A current opinion is that the vascular endotheliature serves as a primary niche for bartonellae. Indeed, bartonellae have been shown to have the ability to induce vasoproliferation, manifesting as verruga peruana (*B. bacilliformis*) or bacillary angiomatosis/peliosis hepatis (*B. henselae* and *B. quintana*). In many in vitro studies, bartonellae have been shown to be able to invade endothelial cell lines and/or interfere with their physiology [24-28] (see below). However, although the endothelial vasculature undeniably plays a role in the early stages of infection, there is some experimental evidence that other putative cell types, such as erythrocytic precursors, may also serve as a niche for infecting bartonellae [29]. This hypothesis, however, conflicts with data obtained using the GFP-*B. tribocorum-*rat model that clearly indicated that encounter with, and invasion of, erythrocytes occurs in the bloodstream [3]. Furthermore, we have been unable to find any evidence for the presence of bartonellae in erythrocyte precursors isolated from the bone marrow of *B. birtlesii*-infected mice, despite rigorous efforts to do so (unpublished observations).

It should also be borne in mind that evidence for vascular endothelial cell involvement in vivo is drawn primarily from pathological observations of *B. bacilliformis*-induced verruga peruana, and bacillary angiomatosis, a rare manifestation of *B. henselae* and *B. quintana* infections of humans. Endothelial cell colonisation has not yet been reported in asymptomatically infected reservoir hosts. Furthermore, pathological study of the vasculoproliferative lesions that characterise verruga peruana and bacillary angiomatosis suggests bartonellae are rather concentrated in proximity to the external surface of the endothelial cells, rather than within them. Thus, although we devote much of this review to the molecular basis of bartonellae-vascular endothelial cell interactions, we do not discount the possibility that an as yet unidentified alternative primary niche may exist.

Exploitation of vascular endothelial cells by bartonellae involves binding to the cell surface, possible internalisation, then persistence within (or adjacent to) cells. To date, two major bacterial factors that play crucial roles in interacting with endothelial cells have been identified, namely the BadA/Vomp/Brp proteins and the VirB/D4 type four secretion system (T4SS) and its effectors. Other bacterial surface proteins, including the haem-binding protein A (Pap31) and Omp43, have also been shown to interact with these cells. All of these factors have also been shown to be essential for the establishment of bacteraemia, as revealed by signature-tagged mutagenesis (STM) screening in both *B. birtlesii*-mouse and *B. tribocorum*-rat infection models, underlying the notion that bacteraemia is not the initial stage of the infection process.

### The badA/vomp/brp adhesions

These proteins have different names in different *Bartonella* species, reflecting their concurrent discovery by independent groups of researchers. These proteins are referred to as bartonella adhesin A (BadA) in *B. henselae*[30], the variably-expressed outer membrane proteins (Vomps) in *B. quintana*[31], and bartonella repetitive protein A (BrpA) in *B. vinsonii*[32]. They are outer membrane proteins belonging to the trimeric autotransporter adhesin (TAA) family [33], that also includes the yersinia adhesin A (YadA) in *Yersinia enterocolitica*[34], the *Haemophilus influenzae* adhesin (Hia) as well as the haemophilus surface fibrils (Hsf) in *H. influenzae*[35], and the ubiquitous surface protein A (UspA) in *Moraxella catarrhalis*[36], all of which are considered to be virulence factors. All TAA family members share similar modular architectures, consisting of a head, neck, stalk, and C-membrane anchor domains [34]. The C-membrane anchor domains define the TAA family and form trimers [37,38]. The size of these proteins varies from one species to another due to the number of TAA neck/stalk repeats, which can differ by up to four-fold. In *B. henselae*, the monomeric form of Bad A is 328kDa in size [30]. In *B. quintana*, Vomps are encoded by a family of four genes, three of which are very similar to *badA* (the exception being *vompD*). Although BrpA remains little studied, the functions of BadA and Vomp have been extensively explored using in vitro assays and experimental animal models. This work has demonstrated the pleiotropy of these proteins, implicating them in (1) mediation of binding of bartonellae to extracellular matrix proteins (collagens and fibronectin) and to endothelial cells, via α-5β-1-integrins (2) circumvention of phagocytosis (3) mediation of angiogenesis via activation of hypoxia-inducible factor 1 (the key transcription factor in angiogenesis) in infected endothelial cells and via provocation of secretion of proangiogenic cytokines (e.g. vascular endothelial growth factor) [30,39]. BadA/Vomps are also involved in bacterial auto-aggregation [31,40], and probably in biofilm formation [41]. Interestingly, there is a marked variation in the expression of BadA between different *B. henselae* strains [42] and it is known that multiple in vitro passage of isolates results in the loss of BadA expression. Different genetic processes, such as single base deletions or insertions, or recombination events, can affect BadA expression resulting in the coexistence of phase variants expressing or not expressing BadA. This characteristic, when coupled with the observation that BadA-expressing strains grow slower than non-expressing strains, suggests that expression of BadA (which is an enormous molecule) is an extremely energetic process, and one which bartonellae will only continue whilst required to do so.

### The VirB/D4 T4SS and its effector proteins

**Figure 2:**
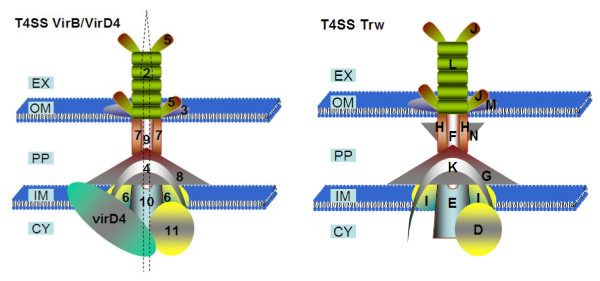
**Hypothetical models of the architectures of the VirB/D4 and Trw T4SS, the VirB/D4 system possesses an inner membrane protein VirD4, the T4CP.** External to the outer membrane, VirB4 and VirB11 energize the secretion process. VirB3, 6, 8, 9 and 10 are thought to form a secretion channel bridging the inner membrane and the surface exposed pilus associated components VirB2 and VirB5. The Bep proteins are secreted via this system. Trw has a similar structure to VirB/D4, with homologs for all VirB proteins except VirD4, which Trw lacks. Unlike VirB/D4, Trw expresses multiple variant copies of TrwL and TrwJ. Abbreviations: EX: extracellular matrix, OM: outer membrane, PP: periplasm, IM: inner membrane, CY: cytoplasm.

T4SSs consist of a multiprotein channel spanning inner and outer Gram-negative bacteria membranes and a surface filament extending from the bacterial envelope. The system mediates the transfer of protein or DNA substrates from a bacterial donor cell into various cell types (e.g. transfer of DNA into other bacteria by conjugation or transfer of bacterial effector molecules into eukaryotic cells). In pathogenic bacteria, this protein complex can be compared to a microscopic syringe that is used to inject effector proteins into target cells in order to subvert their physiology. T4SSs serve as key virulence factors for many important human pathogens including *Helicobacter pyloriLegionella pneumophila, Bordetella pertussis*, and *Brucella melitensis*[43]. Akin to other T4SSs, VirB/D4 is a macromolecular complex of at least 10 components termed VirB2 to VirB11 and an associated substrate recognition receptor known as the T4 coupling protein (T4CP), named VirD4 (Figure 2).

The VirB/D4 T4SS was first identified in *Bartonella* species in 2000 as a result of characterization of the locus that encodes a 17kDa immunodominant protein in *B. henselae*[44]. Genetic comparison of this locus revealed that the 17kDa protein it encoded was a VirB5 homolog and further exploration of the locus revealed the presence of homologs of other members of the T4SS upstream and downstream of VirB5 [44,45]. The putative promoter region of the operon was also identified and its expression was shown to be induced when *B. henselae* was cultivated with human microvascular endothelial cells [46]. The operon was subsequently identified in other *Bartonella* species, and its necessity for host interaction was demonstrated using a *B. tribocorum*-rat and subsequently a *B. birtlesii*-mouse infection model [4,19,47]. Experimentation using these models revealed that although the VirB/D4 system was essential for exploitation of the primary niche, it was dispensable for the subsequent erythrocytic infection [48]. Recent studies have characterised seven genes encoding for effector proteins, termed bartonella effector proteins (Beps) A-G that are translocated by the VirB/D4 into endothelial cells [49] and are responsible for subverting their physiology. Indeed, in vitro experiments have indicated that VirB/D4 and its effector proteins mediate a range of profound changes to parasitised endothelial cells [50,51], including (i) massive rearrangements of the actin cytoskeleton, resulting in the formation and internalisation of large bacterial aggregates by a unique “invasome” structure, (ii) NF-κβ-dependent pro-inflammatory activation leading to cell adhesion molecule expression and chemokine secretion, and (iii) inhibition of apoptotic cell death resulting in enhanced endothelial cell survival. Internalisation of bartonellae via invasomes (characterised by the formation of a bacterial aggregate on the cell surface, which is subsequently engulfed and internalised by an actin-dependant mechanisms [26]) is dependent on three VirB/D4 effectors, BepC, BepG, and BepF [52,53]. BepA has been shown to inhibit endothelial cell apoptosis through upregulation of cAMP levels in the cytosol [49] and it also promotes capillary sprout formation in an endothelial spheroid infection model, whereas BepG inhibits such sprouting [54]. The functions of BepB, BepD and BepE are still to be elucidated. VirB/D4 appears to be part of the regulon of the BatR/S two component regulatory system, a global regulator that may be a key mediator of the physiological transition of bartonellae as they associate with endothelial cells [55].

A homolog of the VirB/D4 system, Vbh, has also been identified and all *Bartonella* species except *B. bacilliformis* possess at least one of these two T4SSs [4]. Comparative genomics have indicated that these systems were acquired by a common ancestor of lineage 2 and 3 *Bartonella* species following its divergence from lineage 1 that carries *B. bacilliformis* (Figure 1) [5]. Given the key roles attributed to these T4SSs, it is clear that its acquisition has resulted in fundamentally different bases of host exploitation by *B. bacilliformis* and the other *Bartonella* species*.* It has been proposed that species possessing the VirB/D4 or Vbh systems have attenuated virulence compared to *B. bacilliformis*, although this view remains controversial. Indeed, given that VirB/D4 and Vbh are so important for endothelial cell interaction in all *Bartonella* species, it is intriguing that the species in which it is absent, *B. bacilliformis*, is the species for which in vivo endothelial cell subversion is the most apparent.

**Figure 3:**
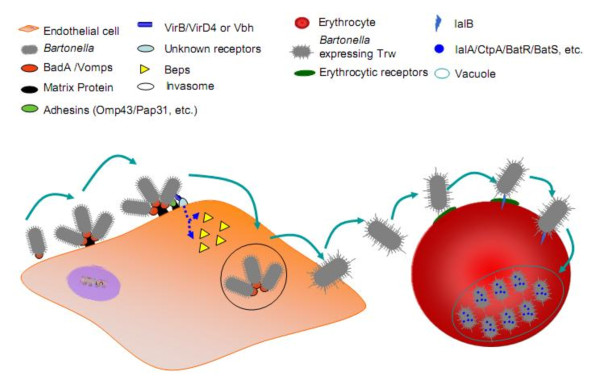
**Holistic view of bartonellae interactions with endothelial cells and erythrocytes.** Exploitation of vascular endothelial cells by bartonellae involves binding to the cell surface via BadA/Vomps proteins as well as VirB/D4 and possible other adhesions as Omp43/pap31. VirB/D4 and its effectors mediate massive rearrangements of the actin cytoskeleton, resulting in the formation of large bacterial aggregates by the invasome structure as well as inhibition of apoptosis leading to enhanced endothelial cell survival. The mechanisms of the passage of *Bartonella* spp. from endothelial cells to erythrocytes is unknown however, it appears that before infecting erythrocytes, the bacteria is free in the blood. Adherence to erythrocytes appears to be mediated by Trw (for most *Bartonella* species) or by flagella (for *B. bacilliformis* and the lineage 2 species). A erythrocyte is usually infected by a single bacterium, which once inside, replicates in a membrane-bound compartment then persists throughout the lifespan of the red blood cell. During this process, proteases IalA and CtpA are suspected to degrade misfolded proteins and to protect bacteria against stress.

Other bartonellae proteins have been shown to interact directly or indirectly with endothelial cells or the extracellular matrix. For instance, the outer membrane lipoprotein Omp43 is one of the bacterial proteins that binds most strongly to human umbilical vein endothelial cells (HUVEC) [56,57]. The Pap31 protein (haem-binding protein A) of *B. henselae* binds to fibronectin and promotes bacterial adhesion to endothelial cells [58]. As for VirB/D4 and Bad/Vomp, the disruption of these genes in the genomes of both *B. birtlesii* and *B. tribocorum* results in the non-appearance of bacteraemia in inoculated animal models [4,19]. Remarkably, *Bartonella* species also appear to secrete the heat-shock protein GroEL, which is a potent mitogen of HUVECs [59]. A holistic view of the synergy between all those virulence factors is schematically represented in Figure 3.

## Step 2: Seeding of blood and extra cellular survival

**Figure 4:**
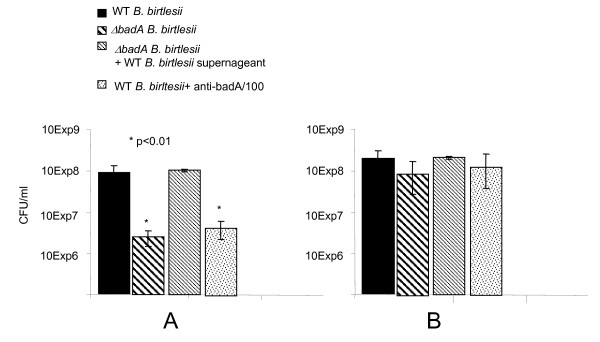
**Role of* B. birtlesii *BadA in complement inactivation.** (**A**) Wild type (WT) and a *badA*-knockout mutant (Δ*badA*) of *B. birtlesii* were incubated with foetal calf serum at 35°C in a 5% CO_2_ atmosphere for one hour then plated onto Columbia blood agar plates. Plates were incubated at 35°C in a 5% CO2 atmosphere for 5 days then colony forming units were counted. N = 6 +/− SE. (**B**) As described in (A) except that foetal calf serum was heated at 56°C for 30 min to inactivate the complement prior to use. N = 6 +/− SE.

**Figure 5:**
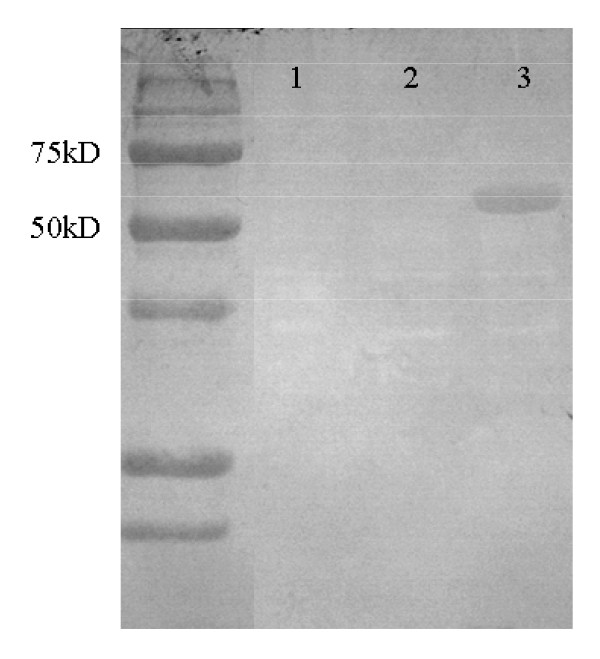
**Far-Western blot of* E. coli *BL21 (1),* E. coli *BL21 transformed with pET30a (Novagen) (2), and* E. coli *BL21 transformed with pET30-AR1 (3) (see below), probed with rat F**_**c**_**fragments conjugated to alkaline phosphatase (Jackson Immunoresearch).** All strains were grown in LB to log-phase and induction of plasmid expression was achieved using IPTG as per the manufacturer’s instructions (Novagen). Construction of pET30a-AR1: The *B. henselae groEL* ORF was amplified using a PCR incorporating primers BhgroELF (AAG GAG AGG AAG AAA TGG CTG CTA AAG AAG T) and BhgroELR (TCA AGG GCT TAG AAA TCC) then cloned into pCR2.1-TOPO and used to transform *E. coli* TOP10 cells according to the manufacturer’s instructions (Invitrogen). The resulting plasmid, pAR1-TOPO, was purified then digested with *Bam*HI and *Xho*I to yield a 1600 bp fragment that included the *groEL* ORF. This fragment was cloned into compatible sites of pET30a (Novagen) to generate pET30-AR1, which was used to transform *E. coli* XL10 cells (Stratagene). pET30-AR1 was recovered from these cells and subcloned into *E. coli* BL21 cells.

In various animal models of infection, bacteraemia appears between 2 and 7 days post infection. It has been proposed that the appearance of bartonellae in the blood is orchestrated, occurring in the form of repeated, discreet waves rather than in a continuous stream [3]. Initially, the bacteria are extracellular, thus their passage must present them with a significant challenge in that they are fully exposed to the host immune system. Recently, we have shown that a *B. birtlesii badA*-knockout (Δ*badA*) mutant was sensitive to mouse serum, while the wildtype *B. birtlesii*, expressing active BadA, was resistant (Figure 4a). Since the Δ*badA* mutant was not killed by heat-inactivated serum (Figure 4b), we suspect that *B. birtlesii* BadA is involved in resistance to complement. In support of this hypothesis, we show that when wildtype *B. birtlesii* is grown in liquid media, the supernatant of this medium has anti-complement activity, but that this activity can be neutralized with anti-BadA antibodies (kindly provided by Professor Volkard Kempf, Goethe-Universität, Frankfurt am Main, Germany). These observations suggest that BadA, or a part of BadA, could be secreted or released by *B. birtlesii* to counter the effects of complement, in a manner akin to that reported for the BadA homolog YadA in *Yersinia enterocolitica*[60]. We have also obtained evidence that bartonellae are capable of binding IgG Fc fragments and hypothesize that by doing so, the bacteria further facilitate their extracellular longevity by subversion of host humoral response. We observed that several *Bartonella* species had the capacity to bind Fc, and most could bind immunoglobins derived from a range of different mammals. Far-Western blotting indicates that the Fc binding capacity was mediated by a protein of approximately 65kDa size, and N-terminal sequencing of this protein demonstrates its identity with the heat shock response protein GroEL. Western blotting of *B. henselae* cellular fractions indicates that GroEL was located in the cytoplasm and in the inner and outer membrane of the cell, as previously demonstrated for *B. bacilliformis*[59]. Expression of recombinant *B. henselae* GroEL conferred an Fc binding capacity on *Escherichia coli* (Figure 5). A further mechanism by which bartonellae may counteract the threat of host immunity is via lipopolysaccharide (LPS) modification. The LPS of *B. henselae* possesses an unusual penta-acylated lipid A with a longchain fatty acid [61], a modification that may attenuate toll-like receptor (TLR) 4-mediated host response to bartonellae endotoxin [61,62].

## Step 3: Erythrocyte encounter and adhesion

The ability of bartonellae to exploit erythrocytes is key to their parasitic strategy and is almost unique amongst bacteria. Once inside erythrocytes, bartonellae occupy an immunologically privileged niche that, by virtue of its position in the circulatory system, facilitates not only persistence within the reservoir host but also uptake by haematophagous vectors.

There is, as yet, no evidence that *Bartonella* species are able to sense and specifically move towards circulating erythrocytes, hence we must currently assume that contact between bacterium and erythrocyte results from their chance encounter. Some *Bartonella* species possess flagella, which may facilitate their movement in blood plasma, but their absence from most species suggests they are not essential appendages and indeed, there is evidence that their value in host interaction occurs elsewhere during the course of infection (see below).

Adherence to erythrocytes by most *Bartonella* species appears to be mediated by the Trw T4SS and as yet uncharacterised receptors. Trw is the third T4SS found in bartonellae, and its importance in the establishment of intra-erythrocytic infections by bartonellae in reservoir hosts has been recognised for some time [63-65]. However, evidence for the direct role of Trw in erythrocyte infection has been only recently obtained following the development of an in vitro model for erythrocyte adherence and invasion [19]. In this study, we identified *B. birtlesii* genes required for erythrocyte infection by identifying, among STM mutants unable to induce bacteraemia in mice, those which could also not invade erythrocytes in vitro. From this screening, we identified nine invasion-deficient mutants. In seven of these, genes within the trw operon were disrupted, whereas in the other two, disruptions were located in the invasion-associated *ialA/B* locus (see below) and in *livG*, a putative ABC-transporter encoding gene. The nature of our screening resulted in the conclusion that the products of all these genes were essential for the adhesion to and/or invasion of erythrocytes rather than replication or persistence within them.

Trw shares homology with the broad-host-range conjugation system of R388 plasmid and was acquired by ancestral bartonellae via horizontal genetic transfer from phylogenetically distant bacterial species [66]. Unlike VirB/D4, the Trw T4SS lacks the coupling protein required for export of effectors, suggesting that it is no longer a secretion system [65]. The *trw* genes of *Bartonella* species are co-linear with the respective genes of plasmid R388 except for the presence of multiple tandem gene duplications of *trwL* (the virB2 homolog) and *trwJ-I-H* (the virB5, virB6 and virB7 homologs). The multiple copies of *trwL* and trw*J* are thought to encode the suface-exposed pilus components of the T4SS, while the products of *trwI* and *trwH* are thought to be involved in pilus elongation and anchorage of the T4SS to the outer membrane (Figure 2). The presence of the multiple copies of these components indicates that they probably participate in the expression of variant pilus forms. It is not known if all *trw* genes are co-expressed and thus if numerous pili variants are concurrently present in the bartonellae population infecting a host or if differential expression of copies of these genes occurs, resulting in different pili variants being present on the bacterial cell surface during different stages of the infection process. It has been hypothesised that the presence of pili variants may facilitate the interaction with different erythrocyte receptors or with the variable forms of a specific receptor found across the breadth of the reservoir host population [64,67].

Our studies have also yielded evidence that Trw is a key determinant of bartonellae host specificity. As discussed above, although bartonellae can infect non-reservoir hosts, when they do so they are unable to establish an intra-erythrocytic bacteraemia. However, we were able to confer on *B. henselae* and *B. quintana* (which are naturally associated with only cats or humans respectively) the ability to interact with rat erythrocytes by transforming them to express the *trw* locus of the rat-adapted species *B. tribocorum*[19].

Even though erythrocyte parasitism is the hallmark of *Bartonella* species, the *trw* genes are not present in all *Bartonella* species, but are restricted to members of lineage 3 with the exception of this lineage’s outlier, *B. clarridgeiae* (Figure 1). This distribution suggests that the Trw T4SS was horizontally acquired by a common ancestor of these members of lineage 3 [4]. Interestingly, the distribution of Trw and flagella among the *Bartonella* species is mutually exclusive (Figure 1), thus it has been proposed that following its acquisition, the function of Trw evolved to replace that performed by flagella. This hypothesis is supported by observations that the flagella of *B. bacilliformis* are involved in adhesion to and entry of erythrocytes [68-70].

## Step 4: Invasion of and persistence within erythrocytes

**Figure 6:**
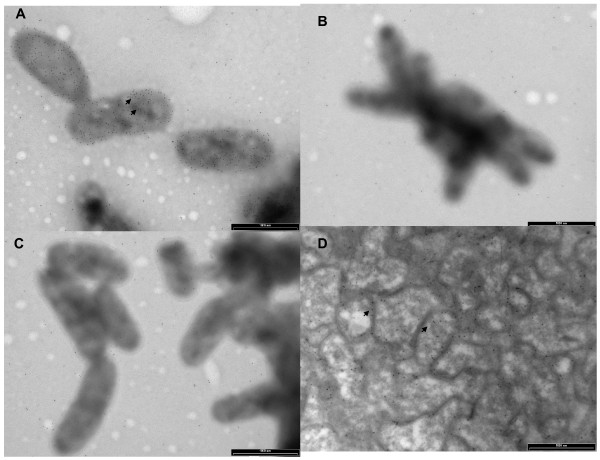
**Localisation of IalB in* B. birtlesii *cells using immunogold staining.** Primary antisera used on intact bacteria were whole cell B. *birtlesii* antiserum (**A**), negative serum (**B**), and *B. birtlesii* recombinant IalB antiserum (**C**). In (**D**) *B. birtlesii* recombinant IalB antiserum was used to localise IalB in cryosections of *B. birtlesii* cells. Arrows indicate the position of immunogold labels in these images.

Among the first virulence factors to be described for bartonellae were those encoded by the *ialA/B* locus in *B. bacilliformis*[71,72]. The survey of *B. birtlesii* genes involved in erythrocyte adherence/invasion, described above, also identified *ialA* and *ialB*. Early work demonstrated that the transformation of *E. coli* with *B. bacilliformis ialA/B* conferred the ability to invade erythrocytes [71] and more recently, we have shown that although deletion of *ialB* did not significantly affect adhesion of *B. birtlesii* to erythrocytes, it provoked a 10 fold decrease in bacterial entry into erythrocytes [19]. IalA has been characterized as a (de)nucleoside polyphosphate hydrolase of the MutT motif family and has homologs in other invasive bacteria such as *Yersinia enterocolitica* and *Rickettsia prowazekii*[73]. The precise role of IalA and its homologs is suspected to be the reduction of stress-induced dinucleotide levels during invasion, thereby enhancing pathogen survival [73]. IalB is a 19.9 kDa protein with about 64% sequence similarity to the *Yersinia enterocolitica* protein Ail, a surface protein that plays a key role in mediating cell entry and serum resistance [60]. Intriguingly, *B. bacilliformis* IalB appears to be localised in the inner membrane [71] so it is unclear how it affects its role in erythrocyte entry. However, more recent work has suggested that in *B. henselae*, the protein is also associated with the outer membrane [72,74]. To add to this uncertainty, we have been able to detect IalB in *B. birtlesii* cryo-sections but not in intact bacteria, supporting the hypothesis that this protein is not surface exposed (Figure 6). Exploration of the determinants of *ialB* expression in *B. bacilliformis* has been reported and the patterns of expression observed under different conditions (temperature, pH, oxidative stress, hemin limitation) suggest that the gene is upregulated in response to environmental cues signalling passage of the bacterium from the vector to the host, and also possibly at times when the bacterium is subjected to stress-inducing environmental conditions [75]. Despite the identification of entry-associated virulence factors, we currently have very little idea about how bartonellae enter erythrocytes. Given the unusual structure and physiology of erythrocytes, it is likely that bartonellae employ a mechanism of invasion that is different from those used for microbial entry into other cell types. How akin this mechanism is to, for example, those employed by *Plasmodium* species for erythrocyte invasion, remains to be seen. Microscopy has been used to monitor *B. bacilliformis* entry into erythrocytes, suggesting that the bacteria provoke then enter substantial deformations in erythrocyte membranes [68]. In this study, bacteria appeared to drive themselves into deep invaginations in the red cell surface, then membrane fusion at the necks of these invaginations led to the formation of intracellular vacuoles containing bacteria [68]. This process was considered, at least in part, to be mediated by the action of flagella. As discussed above, only very few *Bartonella* species express flagella, hence those without these appendages must have evolved an alternative entry strategy. There is also evidence that bartonellae produce an extracellular protein, termed deformation factor, which induces extensive invaginations, dentations and trenches in erythrocyte membranes [76,77].

Intra-erythrocytic replication starts within a vacuolar membrane immediately after invasion [3]. After several days, bacterial replication stops so that the number of intracellular bartonellae remains unchanged for the remaining life span of the erythrocyte. Within the red blood cell, bartonellae must not only scavenge for nutrients and also counter oxidative stress. Genomics has identified potential candidates that may be involved in these processes; proteases IalA and CtpA degrade misfolded proteins that arise from stress, and the genes encoding these proteins have been shown to be essential for the establishment of bacteraemia [4,19]. As yet, none of the molecular mechanisms underlying or regulating multiplication, growth control and then persistence of *Bartonella* species within erythrocytes have been identified.

Akin to many pathogenic bacteria, bartonellae utilizes host haem-containing proteins as a source of haem and iron. It has been shown that *Bartonella* species use a paralogous gene family of haem binding proteins (Hbps) and a haem uptake locus to sequester haem [78-80]. We have previously shown that *B. birtlesii* and *B. tribocorum* strains in which the genes encoding Hbps had been disrupted were unable to induce bacteraemia in their corresponding animal model, indicating the importance of these proteins in host exploitation by *Bartonella* species [4,19]. Both the *hbp* genes and the haemin-associated locus (*hut*) are regulated by Irr, a regulatory protein involved in response to various stimuli including temperature shift, oxygen level and haemin concentration [81,82]. Further studies of those systems will contribute to our understanding of how bartonellae use the most abundant source of haem in the mammalian host, haemoglobin, and this contributes to its persistence within erythrocytes.

The rapid development of transcriptomic technologies should soon provide the means for exploration of the dynamics of bacterial gene expression during host interaction, thus we predict that the transcriptome of intra-erythrocytic bartonellae will soon be available, helping us better understand the molecular means by which bartonellae thrive in their erythrocyte niche.

There is currently no evidence to suggest that infection of erythrocytes by *Bartonella* spp. has a significant effect on their physiology (life span and membrane integrity appear unchanged) [3]. However some subtle changes may occur; for example, during the period of erythrocyte invasion and multiplication, *B. tribocorum*-infected cells are cleared from circulation more rapidly than uninfected cells. However, once intracellular replication has ceased, this difference in clearance rates disappears [3]. These observations suggest that there are “recognizable” changes in erythrocyte structure or physiology during the early stages of their parasitism. These changes may result from the effects of deformin, as discussed above, with altered erythrocytes being filtered out by the spleen as demonstrated for *Plasmodium* spp. [83].

While most *Bartonella* species appear intent on not significantly altering the physiology of the circulating erythrocyte population, *B. bacilliformis* has the potential to provoke severe haemolytic anemia [84], although this pathology is not necessarily a consequence of infection. Evidence that haemolysis may be mediated by a contact-dependant haemolysin has been presented [84], but this protein has yet to be fully characterized. Interestingly, although putative haemolysin-encoding genes are present in the *B. bacilliformis* genome, they are also found in the genomes of other *Bartonella* species.

## Conclusion

The high prevalence of infections in mammals, and the potential threat posed to the health of humans, livestock and companion animals, warrants further exploration of the fundamental biology of *Bartonella* species. Despite a huge effort in the last 20 years to understand the mechanisms used by bartonellae to exploit their natural reservoir hosts, many areas of uncertainty remain and require further research.

In retrospect, one of the key studies that paved the way for recent advances was that completed a decade ago describing the dynamics of”natural” infection, as monitored using fluorescently-labelled bacteria [3]. The identification and quantification of distinct stages of infection, in tandem with the development of reliable tools for the genetic manipulation of bartonellae, has allowed significant advances to be made in our understanding of the molecular basis of bartonellae parasitism. However, despite this progress, and the ever increasing medical importance of bartonellae, it appears that today fewer and fewer scientists are studying these bacteria. It is unthinkable that research into the molecular basis of bartonellae-host interactions should falter now, with so many important and exciting questions still to be answered. For example, what is the fate of bacteria following inoculation? How do bacteria disseminate around the body and is the endothelial vasculature truly a primary niche for infection? Furthermore, why, on occasion, do bartonellae provoke angiogenesis? We also know nothing about how bartonellae regulate their intra-erythrocytic replication and persistence, what physiological changes they endure. We know little about the interaction between bartonellae and host immunity and thus little about the extent and importance of immunoregulation. Researchers also need to consider the role of arthropods in the bartonellae natural cycle and should perhaps incorporate “natural” inoculation by arthropods rather than by syringe into relevant animal models. In summary, the challenges for future years, are (1) to understand how, the unique infection strategy of bartonellae contributes to their remarkable epidemiological success in their reservoir hosts, and (2) to better assess if bartonellae have the potential to emerge as new zoonotic pathogens. In such a small field, a constructive means of helping to invigorate stimulating and high level science is collaboration between those laboratories with expertise in various key technical skills (experimental vector transmission, genetics, animal models) and those with the enthusiasm, but perhaps not all the means, to progress the field. Such initiatives should be encouraged and welcomed by all.

## Competing interests

The authors declare that they have no competing interests.

## Author’s contributions

HKD, RJB, MVT drafted the manuscript. DLR, CV carried out BadA experiments, AR carried out GroEL experiments, HD, MVT performed electron microscopy analysis. All authors read and approved the final manuscript.
